# Role of B Cell Profile for Predicting Secondary Autoimmunity in Patients Treated With Alemtuzumab

**DOI:** 10.3389/fimmu.2021.760546

**Published:** 2021-10-08

**Authors:** Paulette Esperanza Walo-Delgado, Enric Monreal, Silvia Medina, Ester Quintana, Susana Sainz de la Maza, José Ignacio Fernández-Velasco, Paloma Lapuente, Manuel Comabella, Lluis Ramió-Torrentà, Xavier Montalban, Luciana Midaglia, Noelia Villarrubia, Angela Carrasco-Sayalero, Eulalia Rodríguez-Martín, Ernesto Roldán, José Meca-Lallana, Roberto Alvarez-Lafuente, Jaime Masjuan, Lucienne Costa-Frossard, Luisa Maria Villar

**Affiliations:** ^1^ Department of Immunology, Ramón y Cajal University Hospital, Instituto Ramón y Cajal de Investigación Sanitaria (IRYCIS), Red Española de Esclerosis Múltiple (REEM), Madrid, Spain; ^2^ Department of Neurology, Ramón y Cajal University Hospital, IRYCIS, Red Española de Esclerosis Múltiple (REEM), Madrid, Spain; ^3^ Neuroimmunology and Multiple Sclerosis Unit, Neurology Department, Neurodegeneration and Neuroinflammation Research Group, Biomedical Research Institute (IDIBGI), Red Española de Esclerosis Múltiple (REEM), Girona, Spain; ^4^ Servei de Neurologia-Neuroimmunologia, Centre d’Esclerosi Múltiple de Catalunya (Cemcat), Institut de Recerca Vall d’Hebron (VHIR), Hospital Universitari Vall d’Hebron, Universitat Autònoma de Barcelona, Red Española de Esclerosis Múltiple (REEM), Barcelona, Spain; ^5^ Department of Neurology, Virgen de la Arrixaca University Hospital, Murcia, Spain; ^6^ Grupo de Investigación de Factores Ambientales en Enfermedades Degenerativas, Instituto de Investigación Sanitaria del Hospital Clínico San Carlos, Hospital Clínico San Carlos, Red Española de Esclerosis Múltiple (REEM), Madrid, Spain

**Keywords:** multiple sclerosis, side effects, autoimmunity, disease modifying treatments, alemtuzumab, biomarkers, B cells

## Abstract

**Objective:**

To explore if baseline blood lymphocyte profile could identify relapsing remitting multiple sclerosis (RRMS) patients at higher risk of developing secondary autoimmune adverse events (AIAEs) after alemtuzumab treatment.

**Methods:**

Multicenter prospective study including 57 RRMS patients treated with alemtuzumab followed for 3.25 [3.5-4.21] years, (median [interquartile range]). Blood samples were collected at baseline, and leukocyte subsets determined by flow cytometry. We had additional samples one year after the first cycle of alemtuzumab treatment in 39 cases.

**Results:**

Twenty-two patients (38.6%) developed AIAEs during follow-up. They had higher B-cell percentages at baseline (p=0.0014), being differences mainly due to plasmablasts/plasma cells (PB/PC, p=0.0011). Those with no AIAEs had higher percentages of CD4+ T cells (p=0.013), mainly due to terminally differentiated (TD) (p=0.034) and effector memory (EM) (p=0.031) phenotypes. AIAEs- patients also showed higher values of TNF-alpha-producing CD8+ T cells (p=0.029). The percentage of PB/PC was the best variable to differentiate both groups of patients. Baseline values >0.10% closely associated with higher AIAE risk (Odds ratio [OR]: 5.91, 95% CI: 1.83-19.10, p=0.004). When excluding the 12 patients with natalizumab, which decreases blood PB/PC percentages, being the last treatment before alemtuzumab, baseline PB/PC >0.1% even predicted more accurately the risk of AIAEs (OR: 11.67, 95% CI: 2.62-51.89, p=0.0007). The AIAEs+ group continued having high percentages of PB/PC after a year of alemtuzumab treatment (p=0.0058).

**Conclusions:**

A PB/PC percentage <0.1% at baseline identifies MS patients at low risk of secondary autoimmunity during alemtuzumab treatment.​

## Introduction

Alemtuzumab (Lemtrada^®^; Sanofi, Paris, France) is a humanized monoclonal antibody approved for the treatment of relapsing-remitting multiple sclerosis (RRMS). It is administered as two annual courses and proved to be efficacious for patients with highly inflammatory disease, resulting in prolonged remission periods (1).

Alemtuzumab targets CD52, a molecule expressed at high levels by T and B lymphocytes (2). It causes a selective depletion of these cells *via* antibody and complement dependent cytotoxicity and apoptosis (3). T and B-cell repopulation begins within weeks, with a distinctive pattern; B cells undergo faster repopulation, while T cells remain depleted longer (4). Repopulation associates with increases of regulatory CD56bright natural killer (NK) cells (2), and regulatory T cells and with a reduction of the production of pro-inflammatory cytokines (5). This can explain the long duration of clinical effects in the absence of continuous treatment. As a counterpart, frequent adverse events associated with alemtuzumab include infusion-associated reactions, infections, and mainly autoimmune adverse events (AIAEs) (6, 7). The most frequent AIAEs are those involving the thyroid gland, observed in about 38% of patients treated with alemtuzumab (8). Other secondary AIAEs initially described in the clinical trials included immune thrombocytopenia and nephropathies. In the post-marketing setting, new ones have also been reported (9, 10). These side effects, although mild in most cases, limit the use of this drug. Therefore, reliable biomarkers predicting patient individual risk for developing autoimmunity, and hence, guide patient selection for this particular therapy, would be of great clinical importance.

We aimed to explore if the blood lymphocyte profile before alemtuzumab treatment initiation and after a year of treatment could identify MS patients at high risk of AIAEs.

## Materials and Methods

### Study Design

This was a multicenter prospective longitudinal exploratory study including 59 patients diagnosed with RRMS according to 2010 McDonald criteria (11) who were initiating treatment with alemtuzumab. Patients were consecutively recruited in five Spanish hospitals.

### Patients

Patients were followed for 3.55 [3.25-4.21] years (median [25%-75% interquartile range (IQR)]) after alemtuzumab treatment initiation. All patients received two courses of alemtuzumab (12 mg/d IV on five consecutive days at baseline and 12 mg/day IV on three consecutive days 12 month later). Five patients received an additional course for presenting new clinical relapses after the second course. Clinical and demographic data of patients at study inclusion are described in **Table 1**.

**Table 1:** Clinical and demographic features of patients.

Variable	Patients (n = 59)
Sex (F/M)	39/20
Age at disease onset [years] – median (IQR)	28 (23-33.6)
Age at treatment onset [years] – median (IQR)	37 (30-44)
Disease duration [years] – median (IQR)	7.0 (3.0-11.5)
EDSS score at treatment onset – median (IQR)	2.75 (1.5-3.63)
Annualized relapse rate in the two previous years – median (IQR)	0.83 (0.63-1.75)
Previous disease modifying treatments (Yes/No)	46/13
Number of previous treatments - median (range)	2 (0-6)
Last treatment (Number of patients)	
None	14
IFN-beta/GA/Teriflunomide/DMF	19
Fingolimod	13
Natalizumab	13
Time of follow-up since alemtuzumab treatment onset [years] – median (IQR)	3.55 (3.25-4.21)
Alemtuzumab curses (Number of patients)	
Two courses	54
Three courses	5

n, number of patients; F, Female; M, male; EDSS, Expanded Disability Status Scale; IQR, 25%-75% interquartile range; IFN, Interferon; GA, Glatiramer acetate; DMF, Dimethyl fumarate.

### Patient Follow-Up

Patients were evaluated every three months to assess the appearance of new AIAEs, the occurrence of new relapses and the Expanded Disability Status Scale (EDSS) score. MRI scans were performed at baseline and yearly after treatment initiation. Analytical tests, including blood count, serum analyses of renal and thyroid function were performed monthly, while the presence of anti-glomerular basement membrane and anti-thyroid antibodies (including anti-thyroid peroxidase, anti-thyroglobulin and anti-thyroid-stimulating hormone receptor) was tested every three months.

Patients were trained to recognize symptoms suggestive of AIAEs early, and promptly inform the treating physician. AIAEs were defined as the appearance of any autoimmune events during follow-up, with special attention paid to thyroid-associated events, immune thrombocytopenia and autoimmune nephropathy.

### Sample Collection

Blood samples were collected before initiating alemtuzumab and in 39 cases, one year after. Peripheral blood mononuclear cells (PBMCs) were obtained from heparinized whole blood (20 mL) by Ficoll density gradient centrifugation (Abbott) and cryopreserved in aliquots of 5x10 (6) cells until studied. Aditionally, fresh blood was collected in an EDTA tube to explore total cell counts in a Coulter counter.

### Monoclonal Antibodies

Leukocyte subpopulations were assessed using the following monoclonal antibodies: CD3-PerCP, CD3-BV421, CD4-APC-H7, CD8-FITC, CD8-APC-H7, CD14-APC-H7, CD19-PE-Cy7, CD20-FITC, CD24-FITC, CD25-PE-Cy7, CD27-PE, CD38-APC-H7, CD45-V500, CD45RO-APC, CD56-APC, CD127-BV421 and CCR7-PE. Intracellular cytokines were stained using the next panel: Interleukin (IL)-10-PE, Interferon (IFN)-gamma-FITC, Granulocyte-Macrophage Colony Stimulating Factor (GM-CSF)-PE, Tumor necrosis factor (TNF)-alpha-PerCP-Cy5.5 and IL-17A -APC. All monoclonal antibodies were purchased from BD Biosciences, except IL-17A-APC, from R&D Systems.

### Flow Cytometry Analyses

To study surface antigens, cells were stained with the respective monoclonal antibodies during 30 min at 4°C in the dark, washed with saline and analyzed in a FACSCanto II flow cytometer (BD Biosciences), as described before (12).

For intracytoplasmic cytokine detection, cells were stimulated with phorbol-12-myristate-13-acetate (PMA, Merck) and Ionomycin (Merck), in presence of Brefeldin A (GolgiPlug, BD Biosciences) and Monensin (GolgiStop, BD Biosciences), and then incubated 4 h at 37°C in 5% CO2 atmosphere. For the analysis of IL-10 producing B cells, PBMC were incubated prior to stimulation with CpG oligonucleotide (InvivoGen) for 20 h. After incubation, surface markers were stained, then the cellular membrane permeabilized with Cytofix/Cytoperm Kit (BD Biosciences), and incubated with intracellular antibodies, following the same protocol previously described (12).

**Figure 1:**
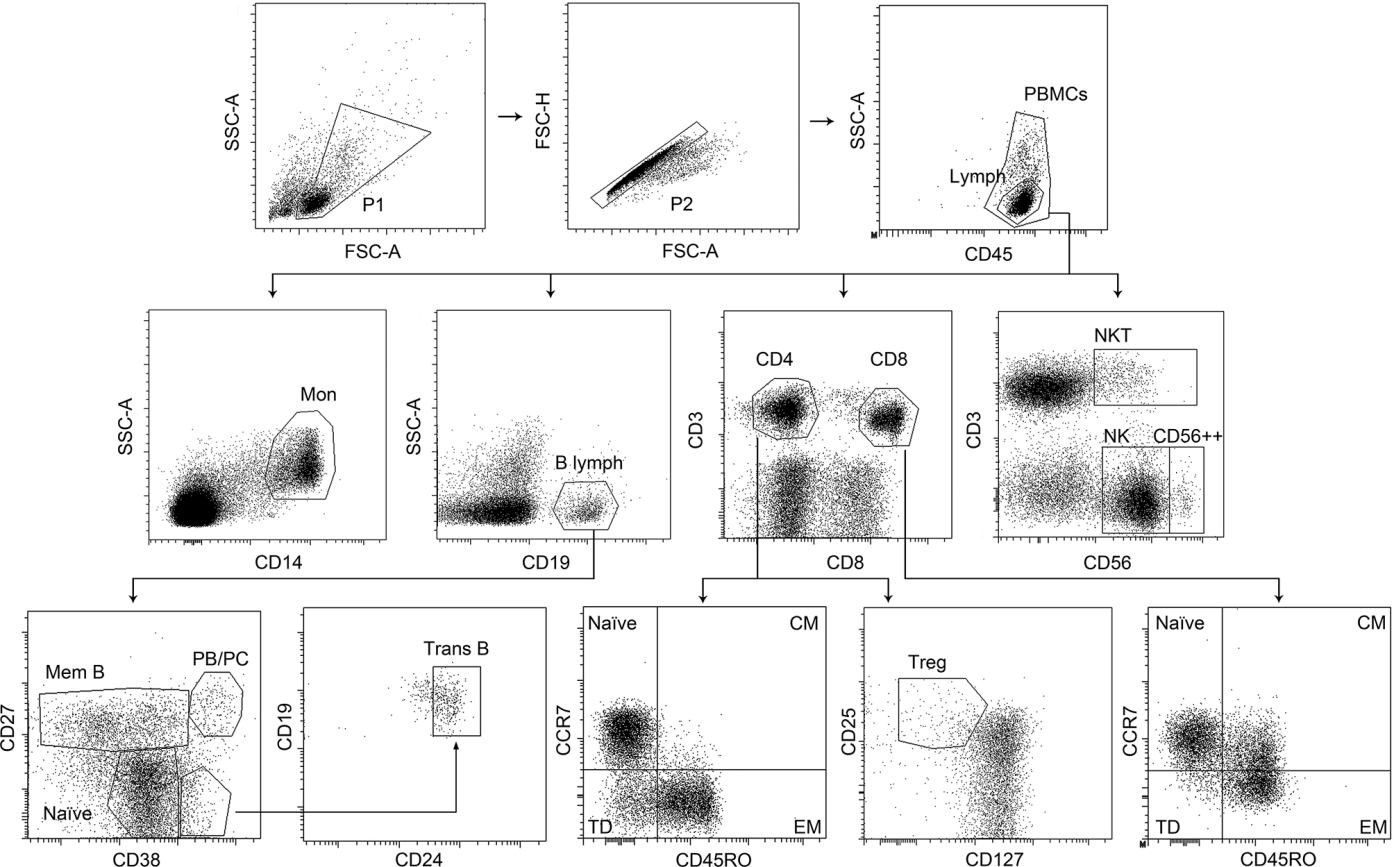
Gating strategy showing representative images for flow cytometry analysis. Peripheral blood mononuclear cells (PBMC) were first gated for excluding debris and apoptotic cells (gate P1) and duplets (gate P2). Lymphocytes were recognized by CD45 staining and low SSC (gate Lymph). Monocytes were recognized as CD14++ cells with intermediate SSC (gate Mon). B cells were detected by the expression of CD19 (gate B lymph) and subdivided by CD27 staining into memory cells: CD27+ (gate Mem B), naïve: CD27- (gate Naïve), plasmablasts/plasma cells: CD27hi, CD38hi (gate PB/PC), and transitional B cells: CD27-, CD38hi, CD24hi, (gate Trans B). Total CD4+ T cells were identified as CD3+ CD8- T cells (gate CD4), and CD8+ T cells were gated as CD3+ CD8++ cells (gate CD8). CD4 and CD8 T cells were classified as naïve: CCR7+ CD45RO− (gate Naïve), central memory: CCR7+ CD45RO+ (gate CM), effector memory: CCR7− CD45RO+ (gate EM), and terminally differentiated: CCR7− CD45RO− (gate TD). CD4+ T regulator cells were identified as CD25hi and CD127-/low (gate Treg). NK cells were identified based on their CD56 expression intensity and CD3 co-expression into CD56+ CD3- (gate NK), CD56+ CD3+ (gate NKT), and CD56++ CD3- (gate CD56++). FSC-A= Forward Scatter-Area; FSC-H= Forward Scatter-Height; SSC-A= Side Scatter-Area.

Cells were analyzed using FACSDiva Software V.8.0 (BD Biosciences). A minimum amount of 5x10 (4) events were acquired. Gating strategy is defined in **Figure 1**.

### Statistical Analysis

Data were analyzed using the Prism 8.0 (GraphPad Software) and Stata 14 (StataCorp) statistical packages. Mann–Whitney U tests were used to explore continuous variables. ROC curves were used to establish cut-off values and Fisher’s exact tests to analyze categorical variables. *p* values below 0.05 were considered as significant.

The missing data were not imputed since the proportion was less than 10% in all variables and we can assume that they were missing completely at random.

### Standard Protocol Approvals, Registrations, and Patient Consents

The study was approved by the ethics committee of the Ramón y Cajal University Hospital. All patients signed a written informed consent form before inclusion.

## Results

Fifty-nine RRMS patients initiating alemtuzumab were included. To avoid bias due to previous autoimmune diseases, one patient was excluded for presenting previous autoimmune pathology. One patient was lost during follow-up. Fifty-seven RRMS patients were finally studied. Twenty-two (38.6%) experienced AIAEs during follow-up. Twenty-one of them presented autoimmune thyroid alterations, and the remaining one developed autoimmune thrombocytopenia. No significant differences were found in clinical and demographic characteristics at baseline or during follow-up between patients showing AIAEs (AIAEs+) and patients who did not develop AIAEs (AIAEs-) (**Table 2**).

**Table 2:** Clinical and demographic characteristics of patients classified according to the appearance of autoimmune adverse events.

Variable	AIAEs+ (n = 22)	AIAEs- (n = 35)	p value
Sex (F/M).	16/6	21/14	ns
Age at disease onset [years] – median (IQR).	26.5 (23-33)	30 (25-36)	ns
Age at alemtuzumab treatment onset [years] – median (IQR)	36 (31-39)	37 (30-47)	ns
Disease duration [years] – median (IQR)	7.0 (1.0-12.0)	7 (3-11.2)	ns
EDSS score at treatment onset – median (IQR).	2.00 (1.5-4.0)	3.0 (2.0-3.5)	ns
Relapse rate in the two previous years – median (IQR)	0.86 (0.51-2.75)	0.78 (0.64-1.61)	ns
Time of follow-up since alemtuzumab treatment onset [years] – median (IQR)	3.78 (3.41-4.59)	3.47 (3.18-3.99)	ns
Number of previous treatments - median (range)	2.5 (0-5)	2.0 (0-6)	ns
Prior treatment (Number of patients)			ns
None (n=14)	7	7	
IFN-beta/GA/Teriflunomide/DMF (n=19)	3	16	
Fingolimod (n=12)	7	5	
Natalizumab (n=12)	5	7	

n, number of patients; AIAEs, Autoimmune adverse events (n=22, consisting of 21 patients with autoimmune thyroiditis, and one patient with autoimmune thrombocytopenia); F, Female; M, male; IQR=25%-75% interquartile range; ns, not significant; EDSS, Expanded Disability Status Scale; IFN, Interferon-beta; GA, Glatiramer acetate; DMF, Dimethyl fumarate. p-values were obtained using Mann–Whitney U tests for continuous variables and Fisher’s exact tests for categorical variables.

### Flow Cytometry Analyses

**Figure 2:**
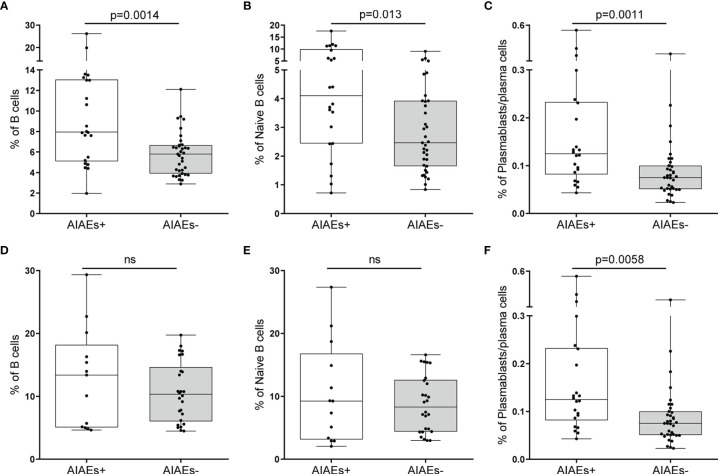
Percentages of cell subsets classified according to the presence or absence of adverse autoimmune events (AIAEs). Percentages of total B cells **(A, D)**, naïve B cells **(B, E)** and plasmablasts **(C, F)** at baseline **(A–C)** and at 12 months of alemtuzumab treatment onset **(D–F)** in 22 patients who developed AIAEs (+) and 35 who did not (-). All percentages are relative to total peripheral blood mononuclear cells. Median values and 25%-75% interquartile range are shown in plots. p-value were obtained using Mann–Whitney U test. ns, not significant.

We studied leukocyte profiles at baseline. Results are shown in **Tables 3** and **4**. When exploring innate immunity, represented by monocytes and NK cells, no significant differences in cellular percentages were observed between AIAEs+ and AIAEs- patients. We did not find differences either in regulatory T and NK as well as in IL-10 producing B and T cells. By contrast, there was an increase in the percentages of CD4+ T cells (p=0.012) in AIAEs- group, mainly due to effector memory (EM, p=0.031) and terminally differentiated (TD, p=0.034) subsets. In addition, AIAEs-patients showed an augment of CD8 T cells producing TNF-alpha (p=0.03). However, the highest differences between AIAEs+ and AIAEs- patients was found in B cells (**Figure 2**). Those acquiring AIAEs showed higher percentages of B cells (p=0.0014), being differences mainly due to plasmablasts/plasma cells (PB/PC, p=0.0011) and to a lesser extent to naïve cells (p=0.013). No significant differences were found in other B cell subsets or in cytokine producing B cells.

**Table 3:** Percentages of peripheral blood mononuclear cells at baseline.

		AIAEs+, n = 22 median (IQR)	AIAEs-, n = 35 median (IQR)	p value
Innate immune cells	NK CD56dim	15.0 (9.58-20.3)	12.2 (8.51-17.7)	ns
	NKT	2.67 (1.58-3.99)	3.30 (1.84-5.80)	ns
	Monocytes	11.3 (8.44-20.1)	13.2 (8.33-21.8)	ns
Regulatory cells	Regulatory T cells	2.08 (1.66–3.34)	2.33 (1.50-3.40)	ns
	NK CD56 bright	0.97 (0.76-1.35)	0.97 (0.55-1.13)	ns
	CD4 IL-10+	0.17 (0.10-0.31)	0.14 (0.10-0.20)	ns
	CD8 IL-10+	0.11 (0.04-0.20)	0.11 (0.06-0.26)	ns
	CD19 IL-10+	0.19 (0.09-0.30)	0.23 (0.10-0.35)	ns
T cells	Total T cells	47.4 (39.7-56.3)	53.3 (48.8-63.8)	0.020
	Total CD4+ T cells	32.4 (26.4-39.9)	41.8 (34.9-45.3)	0.013
	CD4+ Naïve	17.9 (12.7-25.6)	18.9 (10.3-28.5)	ns
	CD4+ CM	8.56 (7.0-10.2)	9.42 (7.76-12.5)	ns
	CD4+ EM	3.83 (3.01-6.64)	7.21 (3.40-11.2)	0.031
	CD4+ TD	1.05 (0.70-1.54)	1.60 (0.93-2.20)	0.034
	Total CD8+ T cells	10.4 (8.14-14.3)	9.19 (6.75-17.1)	ns
	CD8+ Naïve	5.10 (2.93-7.62)	3.27 (1.80-5.60)	ns
	CD8+ CM	0.44 (0.30-0.81)	0.31 (0.24-0.65)	ns
	CD8+ EM	2.22 (1.58-3.35)	1.93 (1.15-3.10)	ns
	CD8+ TD	2.59 (1.45-3.22)	2.40 (0.96-6.40)	ns
B cells	Total B cells	7.95 (5.10-13.1)	5.79 (3.90-6.66)	0.0014
	Transitional B cells	0.09 (0.03-0.20)	0.04 (0.03-0.10)	ns
	Naïve	4.10 (2.44-9.94)	2.46 (1.65-3.90)	0.013
	Memory	2.11 (1.30-3.21)	1.60 (1.25-2.97)	ns
	Plasmablasts/Plasma cells	0.13 (0.08-0.23)	0.07 (0.05-0.10)	0.0011

AIAEs, Autoimmune adverse events; IQR, 25%-75% interquartile range; ns, not significant; NK, Natural Killer cells; NKT, Natural Killer T cells; CM, central memory; EM, effector memory; TD, terminally differentiated. Percentages were calculated over total peripheral blood mononuclear cells. p-values were obtained using Mann–Whitney U test.

**Table 4:** Percentages of T and B cells producing pro-inflammatory cytokines at baseline.

		AIAEs+, n = 22 median (IQR)	AIAEs-, n = 35 median (IQR)	p value
CD4+ T cells	CD4+ TNF-alpha+	13.2 (9.57-17.1)	16.9 (12.9-22.1)	ns
	CD4+ GM-CSF+	1.30 (0.79-1.66)	1.37 (0.97-2.47)	ns
	CD4+ IFN-gamma+	2.60 (2.08-4.50)	3.88 (2.27-5.50)	ns
	CD4+ IL-17+	0.17 (0.09-0.31)	0.12 (0.09-0.20)	ns
CD8+ T cells	CD8+ TNF-alpha+	3.39 (1.98-5.36)	5.42 (3.15-8.02)	0.029
	CD8+ GM-CSF+	0.59 (0.30-1.01)	0.74 (0.50-1.07)	ns
	CD8+ IFN-gamma+	2.48 (1.47-4.05)	3.11 (1.78-4.86)	ns
	CD8+ IL-17+	0.06 (0.03-0.10)	0.09 (0.04-0.10)	ns
B cells	TNF-alpha+	1.70 (1.10-2.99)	2.42 (1.33-5.54)	ns
	GM-CSF+	0.32 (0.18-0.58)	0.30 (0.20-0.40)	ns

AIAEs, Autoimmune adverse events; IQR, 25%-75% interquartile range; ns, not significant; IL, Interleukin; TNF, Tumor necrosis factor; GM-CSF, Granulocyte-Macrophage Colony Stimulating Factor; IFN, Interferon. Percentages were calculated over total peripheral blood mononuclear cells. p-values were obtained using Mann–Whitney U test.

We measured absolute cell counts in all lymphocyte subsets associated with autoimmunity. The relative augment of PB/PC present in AIAEs+ patients was also found when exploring total cell numbers (p=0.024). No significant differences in absolute cell numbers were found in any other cell subset (**Supplementary Table 1**).

**Figure 3:**
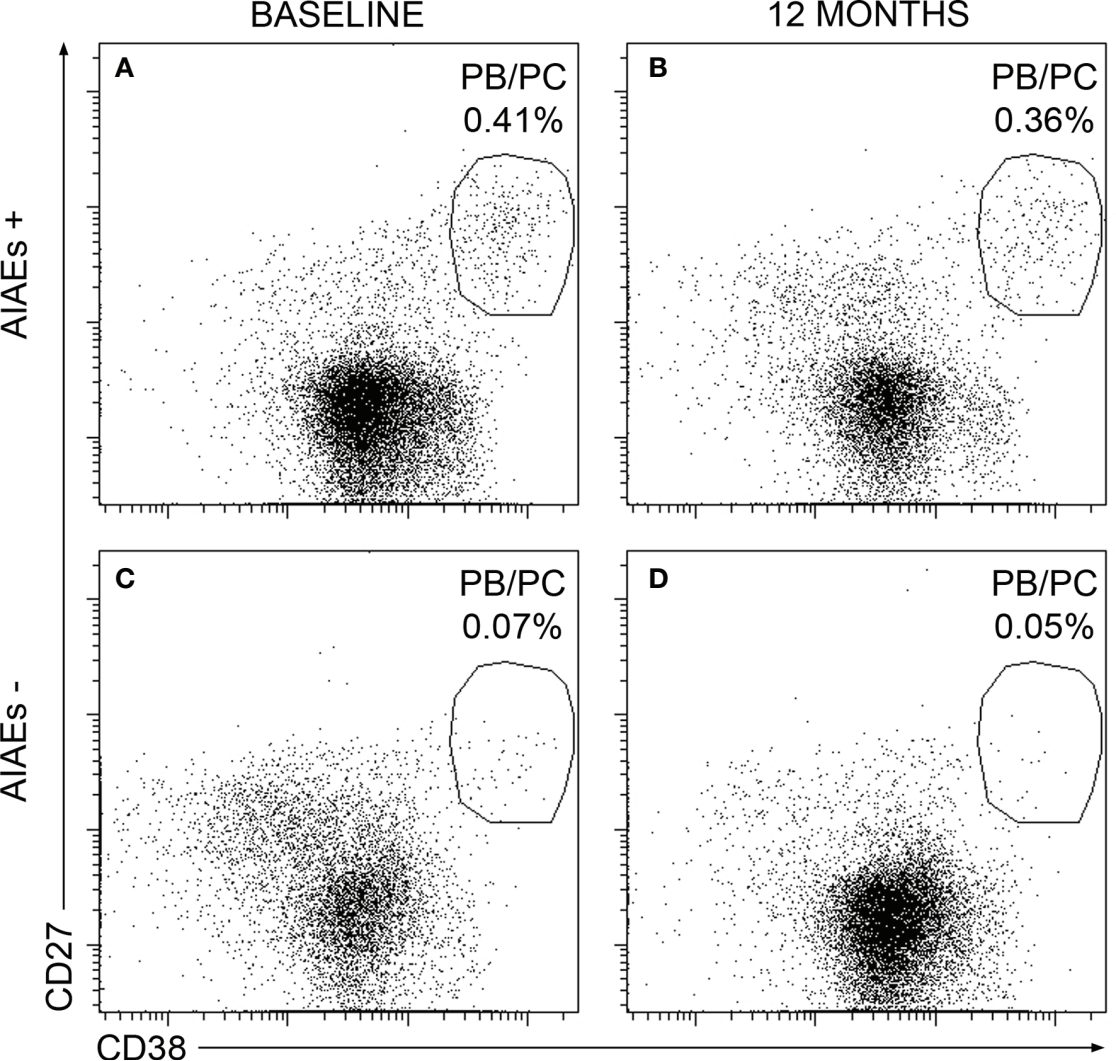
Representative dot plots showing plasmablasts/plasma cells percentages from a AIAEs+ and AIAEs- patient at baseline and after one year of treatment. Percentages of plasmablasts**/**plasma cells from a AIAEs+ patient at baseline **(A)** and after a one year of treatment **(B)** and from a AIAEs- patient at baseline **(C)** and after one year of treatment **(D)**. Percentages are relative to total peripheral blood mononuclear cells.

Finally, we explored if changes observed at baseline remained after a year of alemtuzumab treatment. We studied PBMCs obtained before administering the second cycle of the drug in 39 of the patients studied at baseline (13 of AIAEs+ group and 26 of the AIAEs- one). Results are shown in **Table 5**. PB/PC remained high in AIAEs+ patients a year after administering the first alemtuzumab cycle (p= 0.0058). Representative examples are shown in **Figure 3**. No differences were observed in other cell subsets at this point.

**Table 5:** Percentages of cell subsets one year after first cycle of treatment in patients with secondary autoimmunity who had associations with baseline subsets.

		AIAEs+, n = 13 median (IQR)	AIAEs-, n = 26 median (IQR)	p value
T cells	CD4 T cells	22.0 (17.5-27.4)	18.9 (15.0-21.3)	ns
	CD4 EM	2.55 (2.00-3.67)	4.15 (2.08-6.08)	ns
	CD4 TD	0.70 (0.39-1.19)	0.90 (0.60-1.45)	ns
	CD8+ TNF-alpha	2.30 (1.92-7.64)	2.63 (1.15-3.17)	ns
B cells	Total B cells	13.4 (5.06-18.2)	10.3 (6.00-14.7)	ns
	Naïve	9.25 (3.14-16.8)	8.29 (4.37-12.6)	ns
	Plasmablasts/plasma cells	0.17 (0.09-0.28)	0.09 (0.05-0.10)	0.0058

AIAEs, Autoimmune adverse events; EM, effector memory; IQR, 25%-75% interquartile range; ns, not significant; TD, terminally differentiated. Percentages were calculated over total peripheral blood mononuclear cells. p-values were obtained using Mann–Whitney U test.

**Figure 4:**
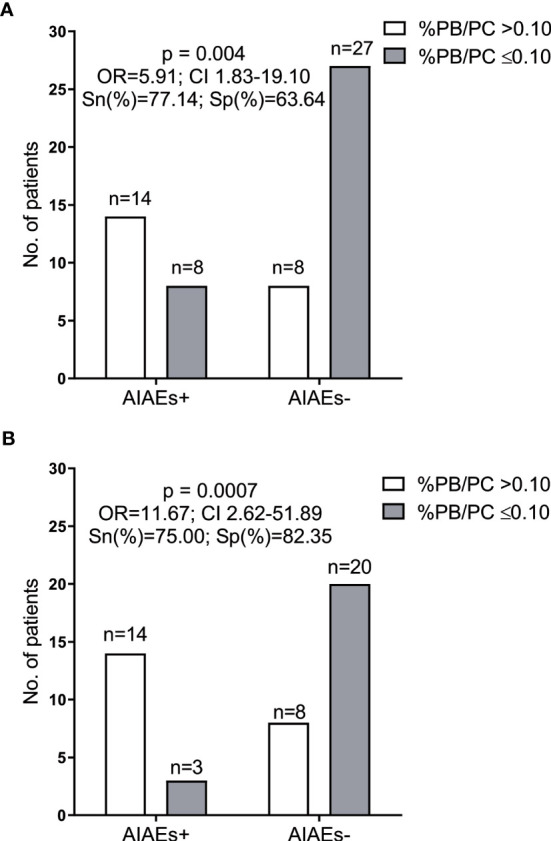
Number of patients with (+) and without (-) autoimmune adverse events (AIAEs) classified according to plasmablasts/plasma cells percentages before alemtuzumab treatment initiation. **(A)** Analysis performed in all patients included in the study (n=57); **(B)** Analysis performed excluding patients who received natalizumab as the last treatment before alemtuzumab (n=45). AIAES, autoimmune adverse events; PB/PC, plasmablasts/plasma cells; N, number; OR, odds ratio; CI, confidence interval. ROC curves were used to establish cut-off values. p-value was obtained using Fisher’s exact tests.

Our data suggest that the percentages of PB/PC at baseline could be a good tool to identify patients with low risk of autoimmunity when treated with alemtuzumab. To address this issue we performed a ROC curve analysis [area under the curve (AUC)=0.75, p=0.001; sensitivity=77.1, specificity=63.6] and established a cut-off value of 0.10. Fourteen out of 22 patients presenting AIAEs but only eight of the 35 AIAEs- patients had a percentage of PB/PC higher than 0.10 [Odds ratio (OR) 5.91, 95% CI 1.83-19.10, p=0.004, **Figure 4A**]. To improve the sensitivity of the assay, we next explored if the heterogeneity found in AIAEs group could be related to previous treatment, since it has been described that natalizumab diminishes the concentration of blood plasmablasts (13, 14). We found that five of the eight AIAEs patients who presented a percentages of PB/PC at baseline <0,1%, received natalizumab as the last treatment prior to alemtuzumab. In fact, PB/PC percentages did not differ in patients previously treated with natalizumab who showed or not AIAEs [AIAEs+ median (IQR)=0.07 (0.06-0.09); AIAEs- median (IQR)=0.05 (0.05-0.07), p=ns]. We explored the effect of the previous treatment with natalizumab in other leukocyte subsets, and did not find any significant changes from our results with the whole cohort.

As a consequence, we performed a new ROC curve analysis on the PB/PC percentages excluding patients who received natalizumab as the last treatment before alemtuzumab. This improved the results (AUC 0.80, p=0.0007; sensitivity=75.0, specificity=82.4). The best cut-off value was again 0.1. Results of the new analysis clearly improved those obtained with the whole cohort (OR=11.67 95% CI 2.62-51.89, p=0.0007, **Figure 4B**).

## Discussion

The search for biomarkers predicting the appearance of new AIAEs after alemtuzumab treatment is of the utmost importance, since these side effects limit the use of this drug, which has proven to be very efficacious in patients with highly active MS (1).

The data obtained in the pivotal phase III trials suggested that the pattern of T- and B-cell depletion and repopulation following alemtuzumab could influence the appearance of secondary autoimmunity (15). However, repopulation kinetics of the peripheral lymphocyte subsets are comparable between patients with or without AIAEs (16).

On the other hand, high serum levels of IL-21, a cytokine promoting T and B-cell differentiation and antibody production, were proposed as predictors of secondary autoimmunity after alemtuzumab treatment (17–19). However ulterior analyses limited its use as a biomarker since there is some overlapping between patients who develop AAIEs and those who not, and detection tests currently available could not distinguish accurately between both groups of patients (20). Likewise, pre-treatment presence of serum anti-thyroid autoantibodies associated with increased risk for clinical onset of thyroid autoimmunity after alemtuzumab treatment (21), but this biomarker would only benefit a small group of patients.

MS is a heterogeneous disease with different immunological mechanisms playing a main role in the inflammatory response (22). Our group focused on studying the immune cell profile before alemtuzumab treatment, assessing both the absolute counts and percentages of the peripheral blood mononuclear cells.

Patients who later developed autoimmunity presented some subtle differences at baseline on T cells, including lower percentages of CD4+ T cells, particularly of terminally differentiated and effector memory cells, and also lower values of CD8+ T cells producing TNF-alpha.

The highest differences were found on B cells, particularly in plasmablasts/plasma cells (PB/PC), which were significantly higher at baseline on patients who later developed autoimmunity. These effector cells, which produce immunoglobulins at a high rate, were implicated in autoimmune responses in MS, where some blood plasmablasts are autoreactive and recognize brain gray matter antigens, what produces and perpetuates an autoimmune response directed toward neurons (23).

When exploring the ability of these cells to predict secondary autoimmunity at baseline, we found that this was not the case in patients treated previously with natalizumab, who mostly presented a lower percentage of plasmablasts. This agrees with previous data showing that natalizumab and no other disease modifying drugs diminish the percentages of plasmablasts in blood (14, 15, 24).

We also observed that those patients who presented at baseline a lymphocyte profile associated with a high probability of developing AIAE, one year after the first cycle of treatment continued presenting high percentages of PB/PC. This may be due to the poor expression of CD52, the target molecule of alemtuzumab, in plasmablasts (25) and mostly in antibody secreting cells (26), which may minimize the effect of this drug in these B cell subsets.

Naïve B cell repopulation is completed about six months after administrating alemtuzumab, while that of CD8+ T cells lasts for a year and reconstitution of memory B cells and of CD4+ T cells and may last more than two years (6, 15). Additionally, the ratios between activated and regulatory cells seem to decrease in patients treated with alemtuzumab during reconstitution (27). This may contribute to the long quality responses reached after the administration of this drug and to the delay in the onset of secondary autoimmunity (6–8). However, when repopulation is completed, the proportion of regulatory T, B and NK cells decreases in patients treated with alemtuzumab (27). The coincidence of lower values of regulatory cells with high numbers of plasmablasts may increase the risk of developing other antibody-mediated autoimmune diseases, as those mainly occurring after alemtuzumab treatment (8). This was observed in our series, where baseline percentages of plasmablasts > 0.10 clearly predicted a higher risk of secondary autoimmunity after alemtuzumab. These data suggest that the immunological mechanisms predominating in individual patients not only influence the response to treatment in MS (28) but can have a clear influence on the side effects.

Although these findings should be validated in larger cohorts with longer follow-up periods, our results suggest that to assess baseline percentages of plasmablasts could be a useful tool to identify MS patients at high risk of autoimmunity upon alemtuzumab treatment, with the limitation of previous treatment, since PB/PC percentages should not be assessed in patients receiving Natalizumab as the last drug before Alemtuzumab initiation.

## Data Availability Statement

The original contributions presented in the study are included in the article/**Supplementary Material**. Further inquiries can be directed to the corresponding author.

## Ethics Statement

The studies involving human participants were reviewed and approved by Ethics committee of the Ramón y Cajal University Hospital. The patients/participants provided their written informed consent to participate in this study.

## Author Contributions

PEWD played a major role in performing experiments, acquisition and analysis of data and drafted the manuscript; EM contributed to statistical analyses and data adquisition. SM, JIFV, PL and NV collected samples and performed flow cytometry experiments; ER and ER-M supervised flow cytometry studies. ACS performed auto-antibody quantification; SSM, MC, EQ, LR-T, XM, LM, JM-L, RA-L, LCF, and JM visited MS patients, contributed to patient inclusion and collected clinical data; LMV designed and supervised the study and revised the draft. All authors revised the manuscript and approved the final version.

## Funding

This work was supported by grants from Red Española de Esclerosis Múltiple (REEM) (RD16/0015/0001; RD16/0015/0004; RD16/0015/0006; RD16/0015/0013) and PI18/00572 integrated in the Plan Estatal I+D+I and co-funded by ISCIII-Subdirección General de Evaluación and Fondo Europeo de Desarrollo Regional (FEDER, “Una manera de hacer Europa”).

## Conflict of Interest

EM received research grants, travel support or honoraria for speaking engagements from Biogen, Merck, Novartis, Roche, and Sanofi-Genzyme. SS received payment for lecturing or travel expenses from Almirall, Biogen, Merck-Serono, Novartis Roche, Sanofi-Genzyme, and Teva. MC has received compensation for consulting services and speaking honoraria from Bayer Schering Pharma, Merk Serono, Biogen-Idec, Teva Pharmaceuticals, Sanofi-Aventis, Genzyme, and Novartis. LR-T has received speaking honoraria and travel expenses for scientific meetings and has participated in advisory boards in the past years with Bayer Schering Pharma, Biogen, EMD Merck Serono, Sanofi Genzyme, Novartis, Sanofi-Aventis, Teva Phramaceuticals, Almirall and Roche. XM has received speaking honoraria and travel expenses for participation in scientific meetings, has been a steering committee member of clinical trials or participated in advisory boards of clinical trials in the past years with Actelion, Alexion, Bayer, Biogen, Celgene, EMD Serono, Genzyme, Immunic, Medday, Merck KGaA, Darmstadt Germany, Mylan, Nervgen, Novartis, Roche, Sanofi-Genzyme, Teva MSIF and NMSS. LM has received travel funding from Genzyme, Roche, Biogen Idec and Novartis, and personal fee for lectures from Roche. JM-L received grants and consulting or speaking fees from Almirall, Biogen, Celgene, Genzyme, Merck, Novartis, Roche and Teva. RA-L reports grants and personal fees from Merck Serono, personal fees and non-financial support from Biogen IDEC, grants, personal fees and non-financial support from Novartis Pharmaceuticals S.A., grants and personal fees from Genzyme, non-financial support from TEVA Pharma, S.L. LC-F received speaker fees, travel support, and/or served on advisory boards by Biogen, Sanofi, Merck, Bayer, Novartis, Roche, Teva, Celgene, Ipsen, Biopas, Almirall. LV received research grants, travel support or honoraria for speaking engagements from Biogen, Merck, Novartis, Roche, Sanofi-Genzyme and Bristol-Myers.

The remaining authors declare that the research was conducted in the absence of any commercial or financial relationships that could be construed as a potential conflict of interest.

## Publisher’s Note

All claims expressed in this article are solely those of the authors and do not necessarily represent those of their affiliated organizations, or those of the publisher, the editors and the reviewers. Any product that may be evaluated in this article, or claim that may be made by its manufacturer, is not guaranteed or endorsed by the publisher.
