# Supplementary material for: Role of B Cell Profile for Predicting Secondary Autoimmunity in Patients Treated With Alemtuzumab
Source: Front Immunol. 2021 Oct 8;12:760546. doi: 10.3389/fimmu.2021.760546 (PMC8531491; doi:10.3389/fimmu.2021.760546)
Supplement: Supplementary file 1 [file Table_1.docx]

**Supplementary Table 1.** Baseline absolute cell counts of the T and B cell subsets associated with secondary autoimmunity.

|  | **AIAEs+, n=22**  **median (IQR)** | **AIAEs-, n=35**  **median (IQR)** | ***p* value** |
| --- | --- | --- | --- |
| T cells | 802.6 (549.7-1031) | 1050 (647.2-1347) | ns |
| CD4+ effector memory cells | 70.8 (48.6-134.4) | 88.4(50.5-187.9) | ns |
| CD4+ terminally differentiated cells | 19.4 (9.85-31.5) | 35.1 (12.2-49.3) | ns |
| TNF-alpha+ producing CD8+ T cells | 76.9 (28.6-105.8) | 88.5 (48.1-165.0) | ns |
| B cells | 126.3 (100.3-191.8) | 116.3 (64.5-146.5) | ns |
| Plasmablasts | 2.40 (1.60-3.35) | 1.40 (0.78-2.50) | 0.024 |

**Footnote to Supplementary Table 1.** AIAEs = Autoimmune adverse events; IQR = 25%-75% interquartile range; ns = not significant.
